# A bidirectional relationship between sleep and oxidative stress in *Drosophila*

**DOI:** 10.1371/journal.pbio.2005206

**Published:** 2018-07-12

**Authors:** Vanessa M. Hill, Reed M. O’Connor, Gunter B. Sissoko, Ifeoma S. Irobunda, Stephen Leong, Julie C. Canman, Nicholas Stavropoulos, Mimi Shirasu-Hiza

**Affiliations:** 1 Department of Genetics and Development, Columbia University Medical Center, New York, New York, United States of America; 2 Columbia University, New York, New York, United States of America; 3 Department of Pathology and Cell Biology, Columbia University Medical Center, New York, New York, United States of America; 4 Department of Neuroscience and Physiology, New York University School of Medicine, New York, New York, United States of America; Washington University in St. Louis, United States of America

## Abstract

Although sleep appears to be broadly conserved in animals, the physiological functions of sleep remain unclear. In this study, we sought to identify a physiological defect common to a diverse group of short-sleeping *Drosophila* mutants, which might provide insight into the function and regulation of sleep. We found that these short-sleeping mutants share a common phenotype of sensitivity to acute oxidative stress, exhibiting shorter survival times than controls. We further showed that increasing sleep in wild-type flies using genetic or pharmacological approaches increases survival after oxidative challenge. Moreover, reducing oxidative stress in the neurons of wild-type flies by overexpression of antioxidant genes reduces the amount of sleep. Together, these results support the hypothesis that a key function of sleep is to defend against oxidative stress and also point to a reciprocal role for reactive oxygen species (ROS) in neurons in the regulation of sleep.

## Author summary

Most animals sleep; humans sleep nearly a third of their lives. Yet the fundamental functions of sleep remain unknown. Here, we used short-sleeping *Drosophila* mutants to uncover a role for sleep in resistance to oxidative stress. Oxidative stress is an imbalance of reactive oxygen species and antioxidant responses. Although these short-sleeping mutants have defects in diverse pathways, they all exhibit sensitivity to oxidative stress. Moreover, increasing sleep in wild-type flies increased resistance to oxidative stress. This suggests that one function of sleep is to defend against oxidative stress. Finally, reducing oxidative stress in neurons of wild-type flies reduces their sleep, suggesting that oxidative stress also regulates sleep. Taken together, our results support an intriguing hypothesis for a bidirectional relationship between sleep and oxidative stress: oxidative stress triggers sleep, which then acts as an antioxidant for both the body and the brain. These results have implications for human patients suffering from chronic sleep restriction and diseases associated with oxidative stress.

## Introduction

A sleeping animal is vulnerable to predators and other dangers in its environment for a large portion of the day. Despite these daily risks, sleep is an evolutionarily conserved behavior throughout the animal kingdom [1–3], suggesting that sleep serves important functions. In support of this, prolonged episodes of acute sleep deprivation in both rodents and invertebrates cause an increased need to sleep [4–7], cognitive impairment [8,9], increased metabolic rate [6,10], and death [6,10,11]. It remains unclear whether these effects are due to loss of sleep or due to the intense stress associated with acute sleep deprivation. Epidemiological studies have revealed that chronic sleep restriction, or shortened sleep duration, in humans is associated with metabolic disorders [12], cardiovascular disease [13], inflammation [14,15], psychiatric disorders [16], and even premature mortality [17,18]. Similar to experimental results involving acute sleep deprivation, it is unclear whether these defects are due to the loss of sleep itself, to associated disruptions in circadian rhythm, or from the very factors that cause sleep loss, such as shift work, aging, or psychological stress. Thus, while current research in both humans and model organisms has demonstrated an important role for sleep in learning and memory [19–22], it has been difficult to identify underlying functions for sleep essential to the organism’s survival or fitness.

Sleep is thought to be regulated by two distinct types of mechanisms: those that control the timing of sleep, such as the circadian system, and those that control the duration of sleep, also called sleep homeostasis mechanisms [23,24]. While the molecular mechanisms underlying circadian regulation have been well characterized, molecular mechanisms regulating sleep homeostasis are less well defined but are thought to be neuronally based [24–29] and context dependent—that is, sleep deprivation or other stress conditions may induce different homeostasis pathways than baseline sleep. Because acute sleep deprivation increases sleep need and results in extended sleep duration at the animal’s next opportunity to sleep, many models of sleep homeostasis propose a feedback mechanism in which the wake state increases sleep-promoting factors, such as adenosine or overall synaptic strength [24,29]. The sleep state then clears or abrogates these factors to allow the wake state.

A controversial hypothesis for the function of sleep is the free radical flux theory of sleep, proposed in a theoretical paper by Reimund in 1994. Reimund proposed that reactive oxygen species (ROS) accumulate in neurons during the wake state and that sleep allows for the clearance of ROS in the brain [30]. ROS are chemically reactive by-products of metabolism, which, when not properly neutralized, cause damaging covalent modifications that inhibit the function of proteins, lipids, and DNA and can lead to cell death. Thus, the free radical flux hypothesis proposed that the core function of sleep is to act as an antioxidant for the brain. Despite the appeal of this hypothesis, data to support it are conflicting. While some groups have reported decreased antioxidant capacity and oxidative damage in the brains of sleep-deprived rats and mice [31–34], other reports have contradicted these findings [35–37]. As a result, the Reimund hypothesis has fallen out of favor as a model for sleep function. Notably, all studies testing the Reimund hypothesis focused on the effects of acute sleep deprivation. In contrast to acute sleep deprivation, the relationship between chronic sleep restriction and oxidative stress has not been thoroughly investigated, despite the physiological relevance of chronic sleep restriction widespread in modern society [38].

In recent years, the fruit fly has become a powerful, genetically tractable model system for the study of sleep [39,40]. Forward genetic screens have identified a number of *Drosophila* mutants that are short sleeping and retain intact circadian rhythms. Loss-of-function mutations in ion channels and ion-channel regulators, including *sleepless*, which regulates the potassium channel Shaker and nicotinic acetylcholine receptors (nAChRs), have been shown to reduce sleep [20,26,41,42]. Other short sleep–causing mutations include the *redeye* allele of the nAChRα4 subunit [43], the *fumin* allele of the dopamine transporter (DAT) [44], and loss of function of the putative ubiquitin ligase adaptor encoded by *insomniac* (*inc*) [45,46]. It has been hypothesized that these mutations cause short sleep by increasing neuronal excitability [24]. These mutants allow researchers to investigate the effects of chronic short sleep independent of circadian defects. While the specific genes affected vary widely and it is not clear whether these mutants sleep less than controls because of reduced sleep need or an inability to sleep, the common phenotype of these diverse mutants is chronic short sleep. Thus, together these mutants provide a system for identifying a “core” or essential function of sleep; we hypothesized that if chronic short sleep has negative effects on health, these diverse short-sleeping *Drosophila* mutants might share a common physiological defect independent of the specific mechanism driving their short sleep.

In this study, we sought to identify a physiological defect common to short-sleeping flies that might provide insight into the function and regulation of sleep. We found that diverse short-sleeping mutants are sensitive to acute oxidative stress, exhibiting shorter survival times than controls, and that increasing total sleep duration of wild-type flies promotes survival after oxidative challenge. We further showed that neuronal overexpression of antioxidant genes in wild-type flies reduces sleep. Our data demonstrate that one function of sleep is to increase the organism’s resistance to oxidative stress and support the hypothesis that sleep abrogates neuronal oxidative stress; these results also point to a role for neuronal ROS in the homeostatic regulation of sleep.

## Results

### Neuronal knockdown of *inc* does not compromise lifespan, metabolism, or immunity

**Fig 1:**
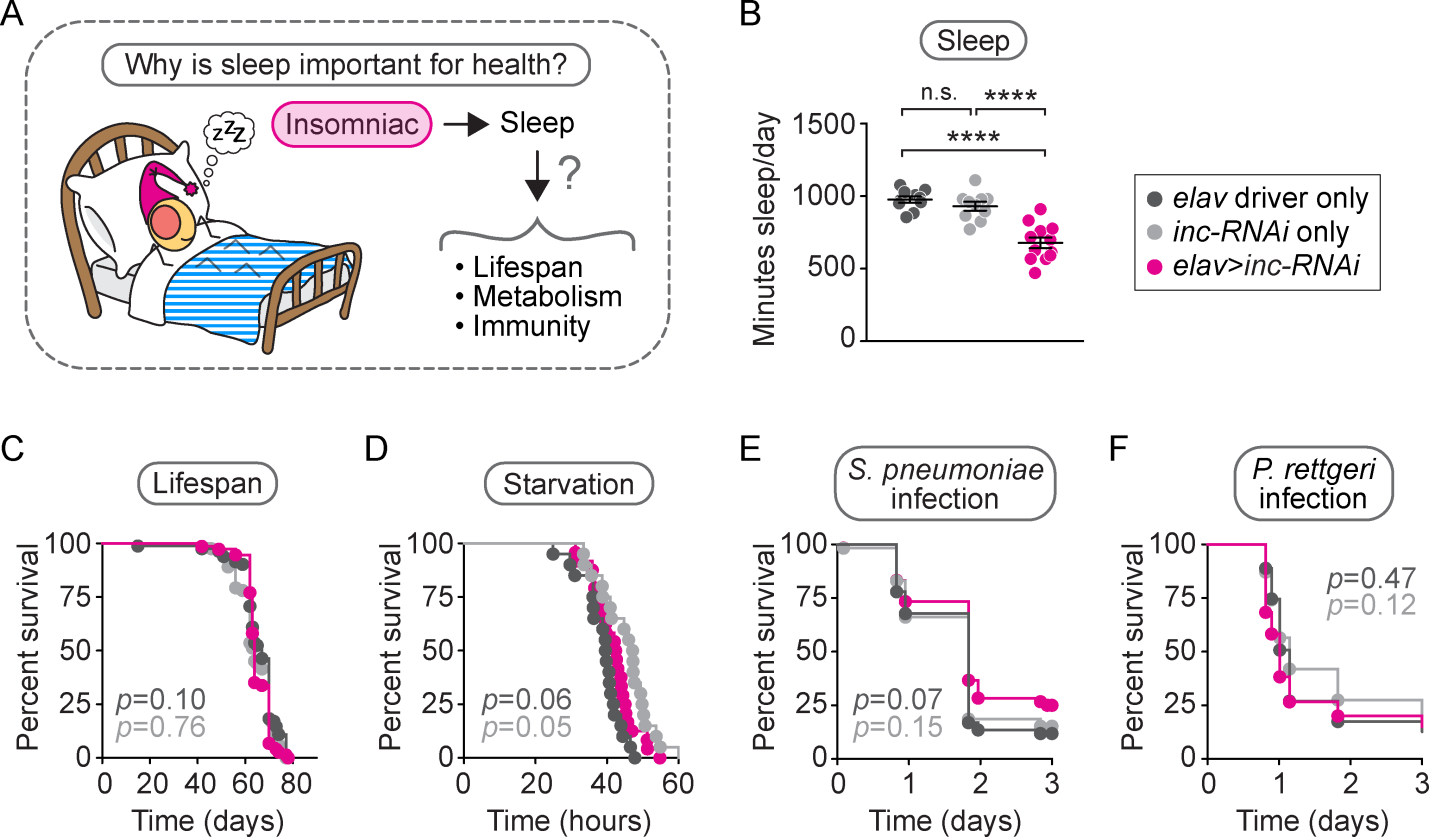
Neuronal *inc-RNAi* reduces sleep without affecting lifespan, metabolism, or immunity. We investigated the importance of sleep in the health of neuronal *inc-RNAi* flies by examining three specific health parameters: lifespan, metabolism, and immunity (A). Relative to genetic controls, neuronal *inc*-*RNAi* flies slept 30% less than controls (B, *p* < 0.0001 compared to either control, *n* = 10–12 flies/genotype), displayed a normal lifespan (C, *p* > 0.05 compared to either control, *n* = 74–82 flies/genotype), died from starvation at an intermediate rate (D, *p* > 0.05 compared to driver control, *p* = 0.05 compared to *inc-RNAi* control, *n* = 20–24 flies/genotype), and died at the same rate as controls after injection with *Streptococcus pneumoniae* (E, *p* > 0.05 compared to either control, *n* = 59–60 flies/genotype) or *Providencia rettgeri* (F, *p* > 0.05 compared to either control, *n* = 60–63 flies/genotype). For the scatterplot in (B), each data point represents the average sleep in minutes/day, measured across 4–5 days for an individual animal. Data are shown as mean ± SEM. *p*-values were obtained by ordinary one-way ANOVA followed by a post hoc Tukey test when significance was detected (B) or by log-rank analysis (C–F). Data from representative experiments are shown. Lifespans were performed twice. All other experiments were performed at least three times. Raw data from representative experiments are available in S1 Data; raw data from all trials are available upon request. *inc*, *insomniac*; n.s., not significant *p* > 0.05; RNAi, RNA interference.

To identify specific physiological functions of sleep (Fig 1A), we first focused on neuron-specific RNA interference (RNAi) of the *inc* gene, which has been shown to cause short sleep [45,46]. *inc* encodes a putative adaptor protein for Cullin-3 (Cul3), an E3 ubiquitin ligase expressed in both the brain and the body. Cul3 is involved in a number of crucial biological processes, and *inc* null mutants have reduced lifespan [45]. In contrast, neuron-specific RNAi of *inc* was reported to cause short sleep without affecting lifespan [45], suggesting that reduction of Inc activity in nonneuronal tissues affects lifespan in a sleep-independent manner. For this reason, we used flies expressing neuron-specific *inc-RNAi* as our initial model of short sleep.

We verified that animals expressing an upstream activation sequence (*UAS*)*-inc-RNAi* construct via the pan-neuronal driver *elav-GAL4*, hereafter referred to as neuronal *inc*-*RNAi* flies, exhibited a 30% reduction in total sleep time relative to isogenic controls carrying one copy of either the *inc-RNAi* construct or *elav* driver alone (Fig 1B, *p* < 0.0001 relative to either control; S1A Fig). We further confirmed that neuronal *inc*-*RNAi* flies exhibit normal lifespan compared to controls (Fig 1C, *p* > 0.5 compared to either control), consistent with a previous report [45] and with recent findings on inbred short-sleeping *Drosophila* lines that have normal lifespan [47]. This result confirms earlier findings that chronic short sleep does not itself shorten lifespan.

Changes in sleep are often associated with altered metabolic energy storage. In humans and mice, sleep loss is associated with metabolic dysfunction such as obesity [48,49], and in flies, starvation suppresses sleep behavior [50] and prolonged sleep is associated with increased starvation resistance [51]. We tested whether neuronal *inc-RNAi* flies have altered starvation resistance, which reflects altered metabolic energy stores. We found that the mortality rate of *inc-RNAi* flies after starvation was intermediate between normally sleeping control flies containing either the *elav* driver or the *UAS-inc-RNAi* construct alone (Fig 1D, *p* = 0.0592 compared to *elav* control, *p* = 0.0493 compared to *inc-RNAi* control), suggesting that short sleep does not affect metabolic energy storage in neuronal *inc*-*RNAi* animals.

Acute sleep deprivation has also been associated with immune dysfunction in humans, rats, and mice [52–55]. Work in flies has shown that acute sleep deprivation can also augment the immune response [56]. To assay for defects or enhancement in immunity because of chronic short sleep, we injected neuronal *inc*-*RNAi* flies with different bacterial pathogens, including *Streptococcus pneumoniae*, a gram-positive pathogen that has been well characterized in *Drosophila* (Fig 1E), *Providencia rettgeri*, a gram-negative natural pathogen found in wild-caught *Drosophila* (Fig 1F), *Listeria monocytogenes*, and *Staphylococcus aureus* (S1B and S1C Fig). In each case, neuronal *inc*-*RNAi* flies died at the same rate as one or both of their genetic controls. To further test whether chronically reduced sleep causes deficits in immune function, we examined the response of short-sleeping *fumin* mutants that lack a functional DAT [44]. We confirmed earlier findings that *fumin* mutants exhibit short sleep (an approximately 95% reduction in sleep relative to controls) (S1D Fig). We found that *fumin* mutants responded variably to these pathogens (S1E–S1H Fig). The lack of a consistent immunity defect across different pathogens in both neuronal *inc*-*RNAi* flies and *fumin* mutants suggests that chronic short sleep does not have a dramatic or common impact on immune function in *Drosophila*.

### Short sleep via reduction of *inc* causes sensitivity to oxidative stress

**Fig 2:**
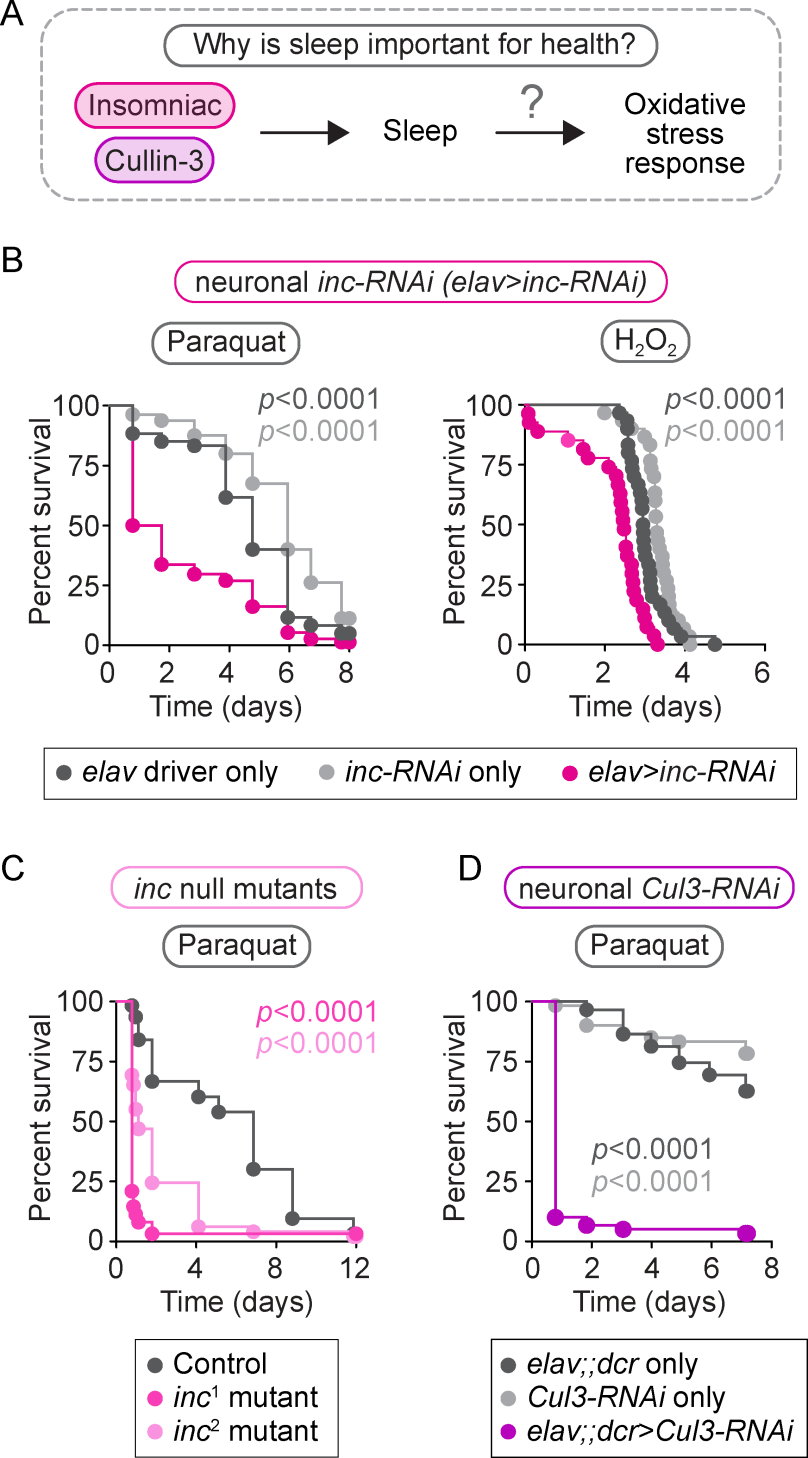
Reducing *inc* or *Cul3* expression results in sensitivity to oxidative stress. We investigated whether reduction of *inc* or *Cul3*, either of which causes short sleep, affects the oxidative stress response (A). Neuronal *inc-RNAi* flies died faster than controls after paraquat injection (B, left panel, *p* < 0.0001 compared to either control, *n* = 60–80 flies/genotype) and H_2_O_2_ feeding (B, right panel, *p* < 0.0001 compared to either control, *n* = 27–30 flies/genotype). Similar sensitivity to paraquat was observed in *inc*^1^ and *inc*^2^ null mutants (C, *p* < 0.0001 for both mutants compared to control, *n* = 49–63 flies/genotype) and neuronal *Cul3-RNAi* flies (D, *p* < 0.0001 compared to either control, *n* = 59–60 flies/genotype). *p*-values were obtained by log-rank analysis. Data from representative experiments are shown. Each experiment was performed at least three times. Raw data from representative experiments are available in S1 Data; raw data from all trials are available upon request. *Cul3*, Cullin-3; *dcr*, UAS-Dicer; *inc*, *insomniac*; *RNAi*, RNA interference.

We next set out to test whether sleep is required to defend against oxidative stress (Fig 2A) [30]. We compared the survival of neuronal *inc*-*RNAi* flies relative to controls when subjected to two different treatments that induce oxidative stress by increasing ROS levels (Fig 2B). We first injected neuronal *inc*-*RNAi* flies with a lethal dose of paraquat, an herbicide that catalyzes the production of superoxide anions [57]. We found that neuronal *inc*-*RNAi* flies died at a significantly faster rate after paraquat injection than controls (Fig 2B, left panel, *p* < 0.0001 relative to either control). To determine whether neuronal *inc*-*RNAi* flies have a specific sensitivity to superoxide anions or if they are also sensitive to other forms of oxidative stress, neuronal *inc*-*RNAi* flies and controls were fed hydrogen peroxide (H_2_O_2_), an oxidant that produces highly reactive hydroxyl radicals and has been shown to alter locomotor activity when fed to flies [58]. Similar to paraquat injection, neuronal *inc*-*RNAi* flies were sensitive to H_2_O_2_ feeding compared to controls (Fig 2B, right panel, *p* < 0.0001 relative to either control). These results indicate that short-sleeping neuronal *inc*-*RNAi* flies are susceptible to oxidative stress.

To verify that oxidative stress sensitivity is caused by the reduction in *inc* expression, rather than an off-target effect of RNAi, we next tested *inc* null mutants for paraquat sensitivity. We confirmed that *inc* null mutants exhibit a 50% reduction in sleep (S2A Fig, *p* < 0.0001 for both *inc*^1^ and *inc*^2^ mutants, relative to controls), as previously reported [45]. Consistent with neuronal *inc*-*RNAi* flies, *inc* null mutants died faster than controls when injected with paraquat (Fig 2C, *p* < 0.0001 for both *inc*^1^ and *inc*^2^ mutants, relative to controls). Furthermore, because Inc is a putative adaptor for the Cul3 ubiquitin ligase, we predicted that reduction of neuronal *Cul3* activity would also cause paraquat sensitivity. As previously reported [45], neuronal *Cul3-RNAi* flies exhibit a 60% reduction in sleep (S2B Fig, *p* < 0.0001 relative to either control); here we found that neuronal *Cul3-RNAi* flies were also sensitive to paraquat injection (Fig 2D, *p* < 0.0001 relative to either control). Thus, chronic short-sleeping *inc* null mutants and *Cul3-RNAi* flies are sensitive to oxidative stress induced by elevated ROS levels, similar to neuronal *inc*-*RNAi* flies.

### Sensitivity to oxidative stress is common to a diverse group of short-sleeping mutants

**Fig 3:**
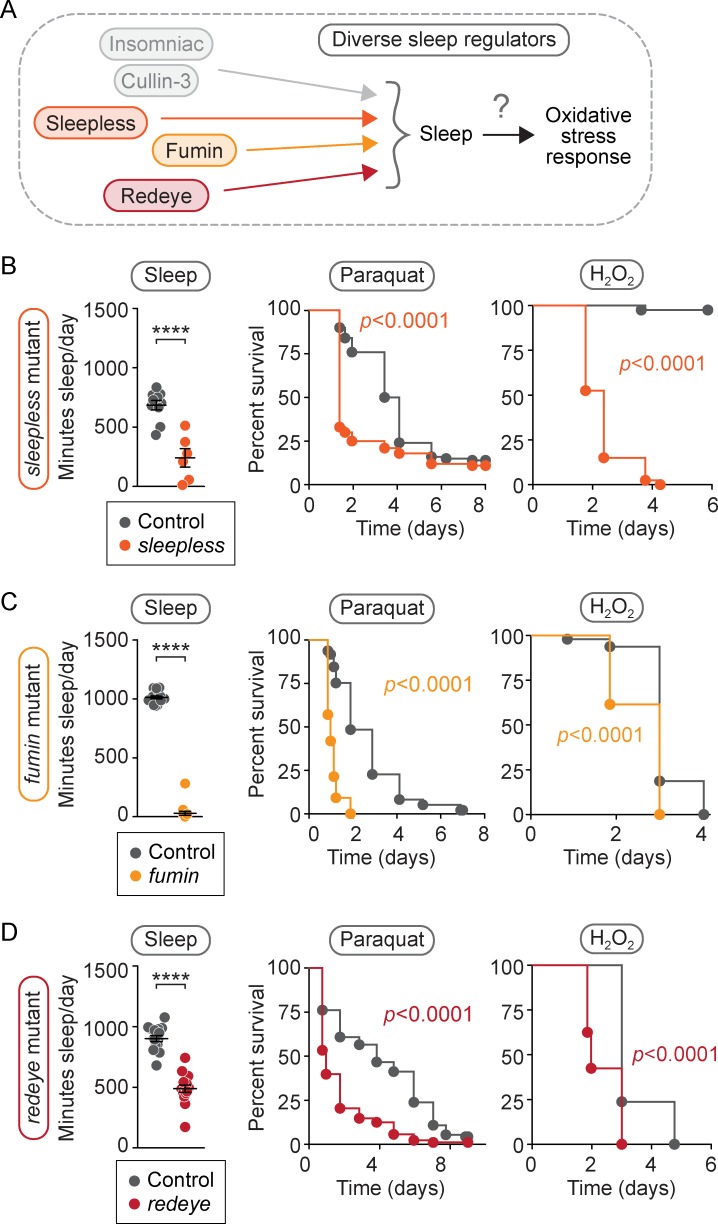
A diverse group of short-sleeping mutants is sensitive to oxidative stress. We asked (A) whether other sleep mutants unrelated to *inc* or *Cul3* share the same sensitivity to oxidative stress. (B–D, left panels) We found that *sleepless* mutants slept 65% less than controls (B, *p* < 0.0001, *n* = 6–10 flies/genotype), *fumin* mutants slept 95% less than controls (C, *p* < 0.0001, *n* = 15–16 flies/genotype), and *redeye* mutants slept 50% less than controls (D, *p* < 0.0001, *n* = 16 flies/genotype). (B–D, middle panels) When injected with paraquat, *sleepless* mutants (B, *p* < 0.0001, *n* = 100 flies/genotype), *fumin* mutants (C, *p* < 0.0001, *n* = 97–98 flies/genotype), and *redeye* mutants (D, *p* < 0.0001, *n* = 88–92 flies/genotype) died faster than controls. (B–D, right panels) Faster death kinetics were also observed after H_2_O_2_ feeding relative to controls for *sleepless* mutants (B, *p* < 0.0001, *n* = 40 flies/genotype), *fumin* mutants (C, *p* < 0.0001, *n* = 39–40 flies/genotype), and *redeye* mutants (D, *p* < 0.0001, *n* = 39–42 flies/genotype). For scatterplots (B–D), each data point represents the average sleep in minutes/day measured across 4–5 days for an individual animal. Data are shown as mean ± SEM and *p*-values were obtained by ordinary one-way ANOVA followed by a post hoc Tukey test when significance was detected. For survival curves (B–D), *p*-values were obtained by log-rank analysis. Data from representative experiments are shown. Each experiment was performed at least three times. Raw data from representative experiments are available in S1 Data; raw data from all trials are available upon request. *Cul3*, Cullin-3; *inc*, *insomniac*.

To determine whether sensitivity to oxidative stress is caused specifically by the reduction in *inc* or *Cul3* activity or whether it is more broadly associated with loss of sleep, we next tested for sensitivity to oxidative stress in three different short-sleeping mutants, each carrying mutations in different genes with varied functions: *sleepless*^Δ40^ (*sleepless*), *DAT*^*fumin*^ (*fumin*), and nAChRα4^*rye*^ (*redeye*) (Fig 3A). We first confirmed, as previously reported [42–44], that each mutant spends significantly less time sleeping than its isogenic control (Fig 3B–3D, left panels, *p* < 0.0001 for each; S1C, S3A and S3B Fig). We next tested these short-sleeping mutants for sensitivity to oxidative stress. Relative to controls, we found that each mutant was sensitive to both paraquat injection (Fig 3B–3D, middle panels, *p* < 0.0001 for each) and H_2_O_2_ feeding (Fig 3B–3D, right panels, *p* < 0.0001 for each). Thus, our finding that this molecularly diverse set of short-sleeping mutants has a common susceptibility to oxidative challenge raises the possibility that sleep itself is required for proper response to oxidative stress.

### Increasing sleep confers resistance to oxidative stress

Because short-sleeping mutants exhibit sensitivity to oxidative stress, we next tested whether extending sleep duration promotes resistance to oxidative stress. We increased sleep by either genetic manipulation or pharmacological treatment and measured the effect on survival after oxidative challenge. For the genetic approach, we used transgenic flies in which sleep-inducing neurons were activated by the expression of a neuron-activating bacterial sodium channel [21]. For the pharmacological approach, we treated wild-type animals with the sleep-inducing drug Gaboxadol [19,59].

**Fig 4:**
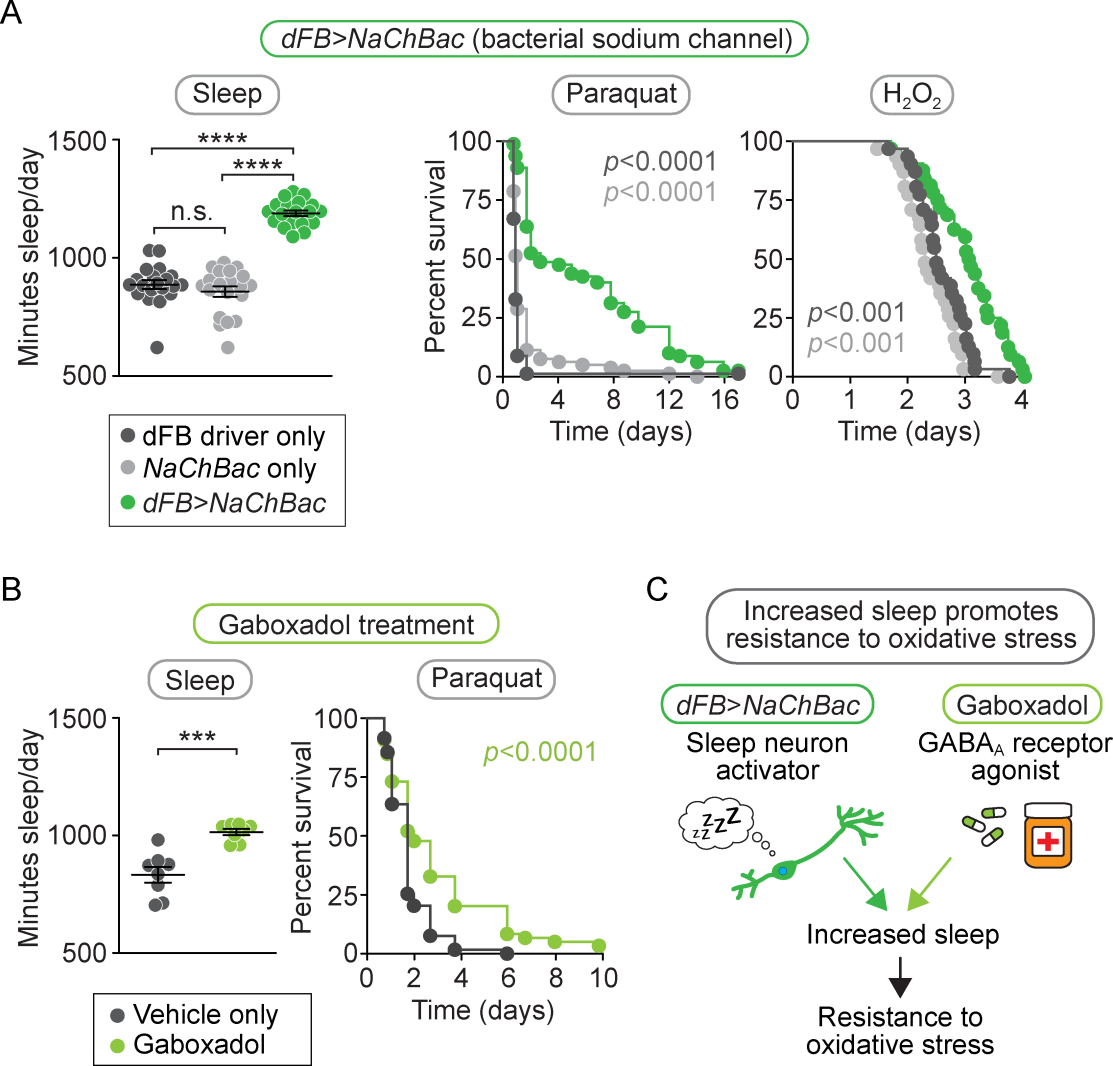
Inducing sleep increases resistance to oxidative stress. (A) *dFB>NaChBac* flies slept 40% more than controls (left panel, *p* < 0.0001 compared to either control, *n* = 20 flies/genotype) and died slower than controls after either paraquat injection (middle panel, *p* < 0.0001 compared to either control, *n* = 79–80 flies/genotype) or H_2_O_2_ feeding (right panel, *p* < 0.001 compared to either control, *n* = 31–32 flies/genotype). (B) Flies fed the GABA_A_ agonist Gaboxadol slept 25% more than controls (left panel, *p* < 0.001, *n* = 8 flies/condition) and died slower than controls after paraquat injection (right panel, *p* < 0.0001, *n* = 118–119 flies/condition). These data support the conclusion (C) that inducing sleep by either genetic or pharmacological means confers oxidative stress resistance. For scatterplots (A–B, left panels), each data point represents average sleep in minutes/day measured across 4–5 days in an individual animal; data are shown as mean ± SEM. *p*-values were obtained by ordinary one-way ANOVA followed by a post hoc Tukey test when significance was detected (A–B, left panels) or by log-rank analysis (A–B, middle and right panels). Data from representative experiments are shown. Each experiment was performed at least three times. Raw data from representative experiments are available in S1 Data; raw data from all trials are available upon request. *dFB*, dorsal Fan-shaped Body; GABA_A_, γ-aminobutyric acid-A.

It was previously shown that total sleep time is increased by constitutively activating neurons in the dorsal Fan-shaped Body (dFB), a sleep-promoting region in the fly brain [21]. We verified this phenotype using a previously established dFB driver (*23E10-GAL4*) [60] to drive expression of the neuron-activating bacterial sodium channel construct *UAS-NaChBac* [61] and observed a 40% increase in sleep duration in *dFB>NaChBac* flies (Fig 4A, left panel, *p* < 0.0001 relative to either control; S3C Fig). We then subjected *dFB>NaChBac* flies to oxidative stress by either paraquat injection or H_2_O_2_ feeding. In both cases, dFB-activated flies died at a slower rate than controls (Fig 4A, middle and right panels, *p* < 0.001 for each). Thus, genetically activating the dFB to increase sleep promotes resistance to oxidative stress.

To further test whether extended sleep duration can increase survival of acute oxidative stress, we used an independent pharmacological method of sleep induction. Wild-type animals were fed the GABA_A_ receptor agonist Gaboxadol, which induces sleep in *Drosophila* [19,59]. We observed a 25% increase in total sleep time in Gaboxadol-treated animals (Fig 4B, left panel, *p* < 0.001; S3D Fig) and a corresponding increase in resistance to paraquat injection relative to vehicle-fed controls (Fig 4B, right panel, *p* < 0.0001). Together, these results demonstrate that two different methods of increasing sleep both promote resistance to oxidative stress, consistent with the idea that oxidative stress resistance is a physiological function of sleep (Fig 4C).

### Neuronal knockdown of *inc* causes altered expression of stress response genes

**Fig 5:**
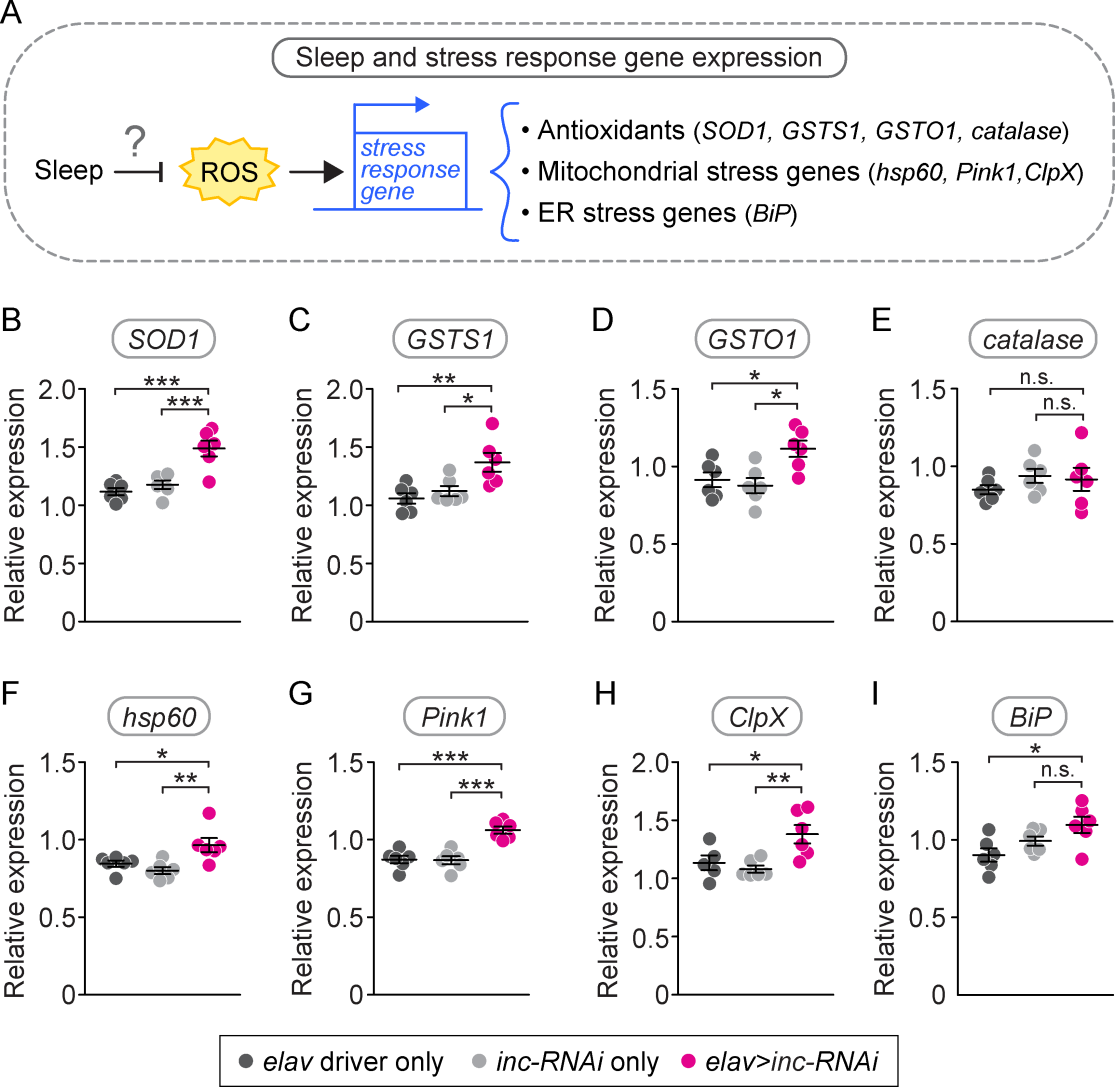
Neuronal *inc-RNAi* heads have increased expression of stress response genes. We investigated whether short sleep affects the expression of three main groups of stress response genes: antioxidant genes, mitochondrial stress genes, and one ER stress gene (A). Neuronal *inc-RNAi* flies exhibited increased baseline head expression of antioxidant genes *SOD1* (B, *p* < 0.001 compared either control, *n* = 6 biological replicates per genotype), *GSTS1* (C, *p* < 0.05 compared to either control, *n* = 6 biological replicates per genotype), and *GSTO1* (D, *p* < 0.05 compared to either control, *n* = 6 biological replicates per genotype), but normal expression of *catalase* (E, *p* > 0.05 compared to either control, *n* = 6 biological replicates per genotype). Neuronal *inc-RNAi* flies also exhibited increased basal head expression of mitochondrial stress genes *hsp60* (F, *p* < 0.05 compared to either control, *n* = 6 biological replicates per genotype), *Pink1* (G, *p* < 0.001 compared to either control, *n* = 6 biological replicates per genotype), and *ClpX* (H, *p* < 0.05 compared to either control, *n* = 5–6 biological replicates per genotype). The ER chaperone gene *BiP* was elevated compared to one, but not both, controls (*p* < 0.05 compared to *elav* control, *p* > 0.05 compared to *inc-RNAi* control, *n* = 6 biological replicates per genotype). Expression was normalized to *actin*. Data are shown as mean ± SEM. Each data point represents an independent biological replicate with 15–20 individual fly heads per biological replicate. *p*-values were obtained by ordinary one-way ANOVA followed by a post hoc Tukey test when significance was detected. Raw data from representative experiments are available in S1 Data; raw data from all trials are available upon request. ER, endoplasmic reticulum; *GST*, glutathione-S-transferase; *hsp60*, heatshock protein 60; *Pink1*, *PTEN-induced putative kinase 1*; *inc*, *insomniac*; *RNAi*, RNA interference; *SOD1*, *superoxide dismutase 1*.

If sleep clears ROS from neurons, one would expect short-sleeping flies to exhibit higher baseline levels of ROS in the brain. Quantitation of ROS in live brains is extremely difficult, possibly due to tight feedback control of ROS levels via the induction of antioxidant gene expression. As an indirect measure of ROS, we measured the expression of genes known to be activated by high levels of ROS by performing quantitative reverse transcription polymerase chain reaction (qRT-PCR) on the heads of neuronal *inc-RNAi* flies and controls (Fig 5A). These genes include the antioxidant genes *superoxide dismutase 1* (*SOD1*), *catalase*, the glutathione-S-transferases *GSTS1* and *GSTO1*, and; the mitochondrial stress response genes *hsp60*, *ClpX*, and *Pink1*; and the endoplasmic reticulum stress response gene *BiP*, which was previously shown to be induced by sleep deprivation [40,62–64]. We found that neuronal *inc-RNAi* flies exhibited increased expression of all of these genes except *catalase* and *BiP* (Fig 5B–5I). While neuronal *inc-RNAi* flies had modestly elevated *BiP* expression in the head (Fig 5I), the difference was not significant. Thus, the increased baseline expression of antioxidant genes and mitochondrial stress genes in neuronal *inc-RNAi* flies is consistent with short sleep causing increased ROS levels in the brain.

### Overexpression of antioxidant genes in neurons reduces sleep

**Fig 6:**
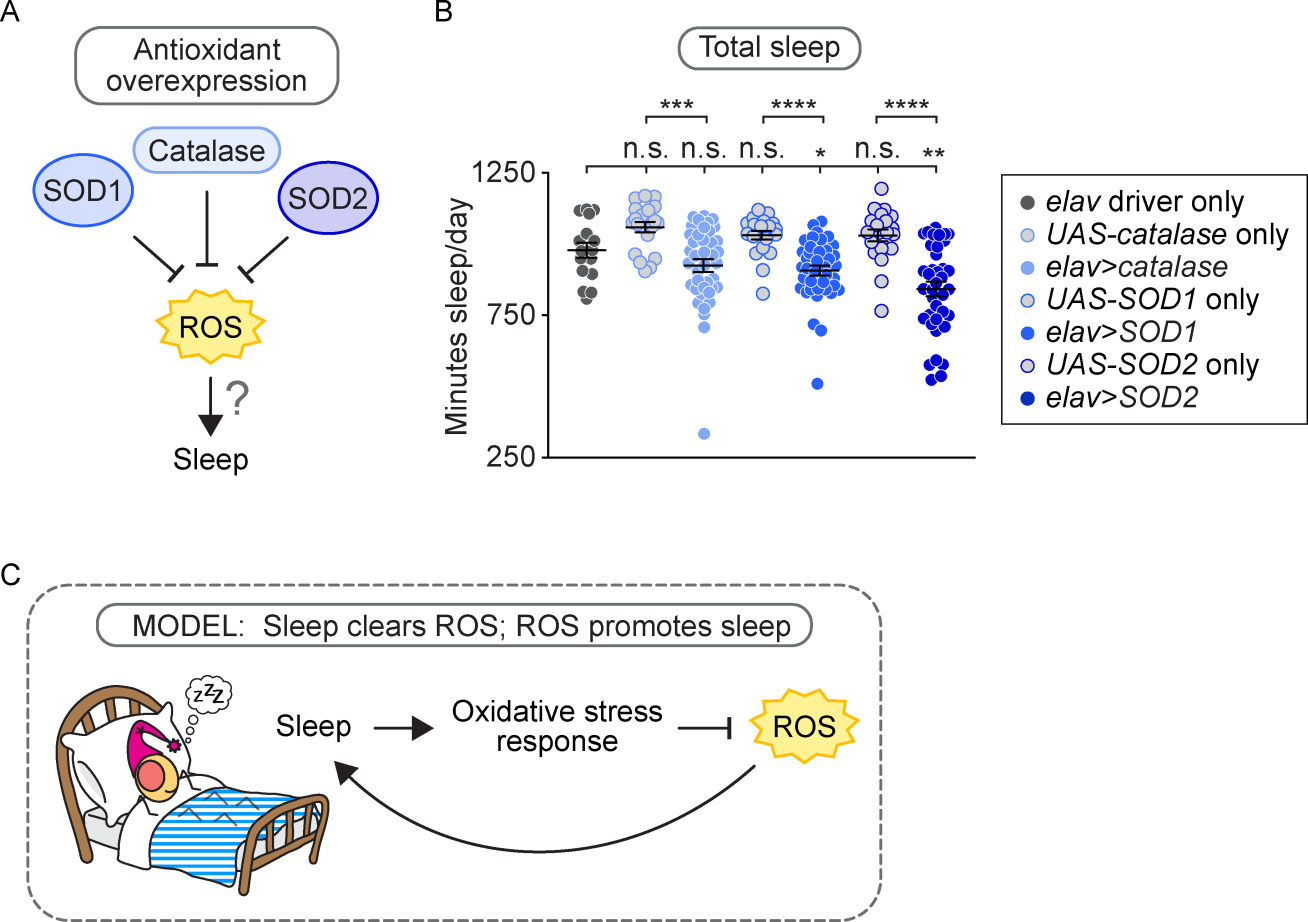
Neuronal overexpression of antioxidants reduces sleep, suggesting a role for ROS in sleep regulation. (A) Neuronal overexpression of the antioxidant genes *SOD1* and *SOD2* reduced sleep by 10% (B, *p* < 0.05 compared to either control, *n* = 16–40 flies/genotype) and 16% (*p* < 0.01 compared to either control, *n* = 16–38 flies/genotype), respectively. Neuronal overexpression of catalase also reduced sleep, but the decrease was not statistically significant compared to the driver control (*p* > 0.05 compared to *elav* control, *p* < 0.001 compared to *catalase* control, *n* = 16–40 flies/genotype). Each data point represents average sleep in minutes/day measured across 5 days in an individual animal; data are shown as mean ± SEM. *p*-values were obtained by ordinary one-way ANOVA followed by a post hoc Tukey test when significance was detected. Pooled data from two independent experiments are shown. (B) Model: high ROS levels promote sleep, which in turn clears ROS to promote wake. Raw data from representative experiments are available in S1 Data; raw data from all trials are available upon request. ROS, reactive oxygen species; *SOD*, *superoxide dismutase*.

If one function of sleep is to clear ROS from the brain, then it is plausible that ROS itself may be a factor that triggers sleep, perhaps when it reaches a certain critical threshold. To determine whether neuronal ROS levels play a role in the regulation of sleep, we reduced ROS levels in the brains of otherwise wild-type flies by driving neuronal overexpression of the antioxidant genes *catalase*, *SOD1*, or *SOD2* using the *elav-Gal4* driver (Fig 6A). *SOD1* or *SOD2* overexpression resulted in a significant reduction in the total amount of sleep, with an average decrease in total sleep of 10% and 16%, respectively (Fig 6B, *p* < 0.05 compared to either control; S3F and S3G Fig). *catalase* overexpression resulted in a similar trend but did not reach significance compared to the driver control (Fig 6B, S3E Fig). Our observation that reducing neuronal ROS levels reduces sleep amount suggests that ROS levels reflect sleep need and play a role in the regulation of sleep (Fig 6C).

## Discussion

Although sleep appears to be evolutionarily conserved across all animal species [1–3], the physiological function of sleep remains unclear. Our data show that chronic sleep restriction sensitizes flies to two types of oxidative stress: paraquat injection and H_2_O_2_ feeding (Figs 2 and 3). Conversely, increasing sleep through either genetic or pharmacological methods promotes resistance to oxidative stress (Fig 4). Thus, our data suggest that one important function of sleep is defense against oxidative stress.

The molecular mechanisms underlying the susceptibility of short-sleeping mutants to acute oxidative stress and whether this susceptibility is due to the effects of oxidative stress on the brain or other, nonneuronal tissues of the body remains unclear. It is possible that increased baseline ROS levels in neurons or other tissues sensitize short sleepers to acute oxidative stress. Other investigators have found that accumulation of cellular ROS was associated with susceptibility to acute oxidative challenge [65,66]. Chronic sleep loss may lead to accumulated mitochondrial damage that, in the presence of an acute oxidative stress, triggers cell death pathways. Another possibility is that short sleepers are less able to detect or respond to acute oxidative challenge in specific tissues. Testing these hypotheses will be an important focus for future investigation.

Our data also suggest that short-sleeping animals accumulate higher baseline ROS levels in the brain. While ROS levels in the brain are difficult to measure directly, we observed increased expression of antioxidant and mitochondrial stress response genes in the heads of short-sleeping neuronal *inc*-*RNAi* flies, consistent with increased ROS levels in the brain. Other studies have similarly observed that sleep-deprived animals display increased expression of genes induced by high ROS levels. Induction of the antioxidant regulator *cap’n’collar* (*cnc*) was observed in fly heads when flies were exposed to recurrent sleep fragmentation [67], and its mammalian homolog *Nuclear factor (erythroid-derived 2)-like 2* (*nrf2*) was reported to be induced in the cerebral cortex of mice after 6 hours of sleep deprivation [68]. Sleep deprivation has also been associated with activation of the unfolded protein response in the ER in fly heads and mouse brains [40,62–64]. Because both the ER- and mitochondrial unfolded protein responses can be induced by high levels of ROS, we hypothesize that both genetic and environmental sleep loss increase baseline ROS levels that, depending on the specific method of sleep deprivation, genetic background, and tissue tested, are reflected in the activation of different response pathways.

Finally, we found that increasing antioxidant gene expression in the brain causes short sleep, suggesting that decreasing neuronal ROS levels will promote the wake state. Emerging evidence demonstrates that ROS can act as crucial signaling molecules in a number of biological processes [69,70], and it has been demonstrated that injecting an oxidant into the rat brain induces sleep [71]. One study showed modest effects of lifelong, low-dose paraquat feeding on sleep in flies [72]. Thus, ROS levels, either directly or indirectly through the activation of oxidative stress responses, appear to induce sleep.

Taken together, our results support a model for a bidirectional relationship between sleep and oxidative stress, in which one function of sleep is to act as an antioxidant for both the body and the brain, increasing the organism’s resistance to acute oxidative challenge and reducing ROS levels in the brain; moreover, neuronal ROS play a role in the regulation of sleep and wake states (Fig 6C). Thus, with chronic sleep restriction, the animal accumulates higher ROS levels in the brain and is sensitive to acute oxidative stress.

Identifying the physiological functions and key regulators of sleep is critical to understanding the negative effects on health associated with chronic sleep restriction. In the United States, average sleep time is steadily decreasing [73], and one third of adults sleep less than the recommended 7 hours per night [38]. Sleep restriction is correlated with a variety of diseases [12,13], many of which are also associated with oxidative stress [74–78]. Sleep disturbances have been implicated as a predictor for Alzheimer, Parkinson, and Huntington’s diseases [79–82], and in all of these diseases, oxidative damage has been reported in the brains of patients postmortem [83–85]. Because oxidative stress can induce protein misfolding and aggregation through protein damage, neuronal accumulation of ROS is a plausible contributing factor in the pathogenesis of neurodegenerative diseases. Thus, understanding the role of sleep in defense against oxidative stress and the role of ROS in regulating sleep could provide much-needed insight into the pathology and treatment of neurodegenerative diseases.

## Materials and methods

Results from all experiments are summarized in S1 Table in the Supporting information, and raw data are available upon request.

### Fly strains and rearing conditions

The following flies were used to manipulate *inc* and *Cul3* as described previously [45]: *UAS-inc-RNAi* (VDRC stock #18225), *elav*^C155^*-Gal4*, *UAS-Dicer* (dcr) (Bloomington stock #24651), *inc*^1^ deletion mutant, and *inc*^2^ transposon insertion mutant (CG32810^f00285^), all in the same genetic background (w^1118^ iso31 or Bloomington stock #5905), along with the isogenic iso31 strain used for outcrossing. *UAS-Cul3-RNAi* (NIG stock #11861R-2) was in the NIG *w*^1118^ background and compared to its isogenic control. For neuronal Cul3 knockdown experiments, the *UAS-Dicer* line (Bloomington stock #24651) was crossed into the *elav*^C155^*-Gal4* line. Parental controls used for experiments were obtained by crossing expression driver (e.g., *elav-Gal4*) and RNAi construct (e.g., *UAS-inc-RNAi*) lines to the outcrossed wild-type line (e.g., iso31) for heterozygous controls, accounting for differences in complex phenotypes affected by genetic background. In case the absence of the *white* gene, which encodes an ABC transporter, has an effect on survival after paraquat or H_2_O_2_ exposure, red-eyed controls were used with the red- and orange-eyed *inc*^1^ and *inc*^2^ mutants; these *w*^*+*^ controls were generated by outcrossing *w*^*+*^ from an Oregon-R background for eight generations with the *iso31* stock (Bloomington stock #5905).

*redeye*, *sleepless*^Δ40^ (imprecise excision mutants), and their corresponding background-matched controls were obtained from Amita Sehgal (University of Pennsylvania). *sleepless*^Δ40^ was used instead of *sleepless*^P1^ because *sleepless*^P1^ flies were sensitive to CO_2_, which made paraquat injection experiments difficult to interpret. Male *sleepless*^Δ40^ flies also exhibited some wounding sensitivity, whereas females did not, so female *sleepless*^Δ40^ flies were used in the paraquat injection experiments (S4 Fig). Male *sleepless*^Δ40^ were used in H_2_O_2_ feeding experiments. *fumin* mutants and their background-matched controls were obtained from Rob Jackson (Tufts University).

*UAS-NaChBac* [61] was obtained from Paul Shaw (Washington University, St. Louis, MO) and *23E10-Gal4* [60] was obtained from Jeffrey Donlea (University of Oxford); both were outcrossed for eight generations with the iso31 stock. As described above, parental controls used for experiments were obtained by crossing expression driver (*23E10-Gal4*) and transgene construct (*UAS-NaChBac*) lines to the outcrossed wild-type line (iso31) for heterozygous controls.

The following stocks were obtained from the Bloomington Stock Center (BDSC, Bloomington, IN) and outcrossed 6–8 generations into the iso31 background: *UAS-SOD1* (#24754), *UAS-SOD2* (#24492), and *UAS-cat* (#24621).

All flies were raised at room temperature on standard molasses food (5.85% cornmeal, 2.675% yeast, 0.575% agar, 3% v/v blackstrap molasses, 0.14% methylparaben, 0.5% v/v propionic acid) and kept on cornmeal food (4% cornmeal, 2.15% yeast, 9% dextrose, 0.75% agar, 0.095% methylparaben) post-eclosion in a temperature- (25°C) and humidity- (55%) controlled incubator with a 12-hour light–dark cycle. Four- to ten-day-old males were used for all experiments, unless otherwise noted.

### Sleep analysis and starvation assay

Individual flies were loaded into plastic tubes containing cornmeal food and allowed to acclimate for 1 day. Sleep was monitored for 4–5 days using *Drosophila* Activity Monitors (either DAM2s or DAM5s, an older model of DAM5M with a single beam per tube) (Trikinetics, Waltham, MA). Activity was recorded as beam-breaks in 1-minute bins and analyzed using PySolo software [86] or Microsoft Excel, with sleep defined as a 5-minute period of inactivity. Graphing and statistical analysis were performed using GraphPad Prism (survival assays and scatterplots) and PySolo (24-hour sleep profiles). When comparing two groups: an unpaired *t* test was performed when standard deviations were similar, and an unpaired *t* test with Welch’s correction was performed when standard deviations were not similar (F test *p* < 0.5). When comparing three groups, a one-way ANOVA was performed and followed by a post hoc Tukey test to compare means when significance was detected.

For starvation assays, flies were transferred to tubes containing 1% agar and loaded into *Drosophila* Activity Monitors. Time of death was determined by complete loss of movement.

### Lifespan

Flies were collected on the day of eclosion and allowed to mate overnight. Total flies per genotype ranged from 74 to 225. Numbers were roughly equivalent for each group within different trials. Males were separated into groups of 20 per vial. Flies were transferred to new vials every 2–7 days and scored for death at time of transfer. Lifespan experiments were performed in at least two independent trials.

### Bacterial and paraquat injections

Injections were carried out with a pulled glass capillary needle. A custom-made microinjector (Tritech Research, Los Angeles, CA) was used to inject 50 nL of liquid into the abdomen of each fly. Volume was calibrated by measuring the diameter of the expelled drop under oil. Death was assayed visually at least daily, with a typical *n* = 60 for both bacterial infections and paraquat injections. For each experiment, a smaller set of flies was injected with vehicle alone to ensure that wounding caused minimal death.

The following bacterial strains were used for injections: *S*. *pneumoniae* (strain SP1, a streptomycin-resistant variant of D39) obtained from Elizabeth Joyce (University of California, San Francisco, CA) was grown standing in Brain Heart Infusion media (BHI, Teknova, Hollister, CA) at 37°C with 5% CO_2_, frozen into aliquots with 10% glycerol, pelleted and resuspended upon thawing, and injected at an OD_600_ of 0.015–0.05; *P*. *rettgeri* (strain Dmel, a natural pathogen isolated from wild-caught *D*. *melanogaster* [87]) obtained from Brian Lazzaro (Cornell University) was grown shaking in LB at 37°C and injected at an OD_600_ of 0.003–0.005; *L*. *monocytogenes* (strain 10403S) obtained from Julie Theriot (Stanford University) was grown standing in BHI at 37°C and injected at an OD_600_ of 0.075–0.2; and *S*. *aureus* strain 12600 (ATCC) was grown shaking in BHI at 37°C and injected at an OD_600_ of 0.0001–0.001. Postinjection, flies were kept in a 29°C incubator for the remainder of the experiment to allow for optimal infection, with the exception of *P*. *rettgeri* injection, in which case optimal infection was achieved at 25°C. All OD_600_ measurements were made using a Genesys 10S Vis Spectrophotometer (ThermoScientific, Waltham, MA), blanked against the corresponding sterile media for the given culture. Cultures were then diluted in sterile media to the desired OD.

For paraquat injections, paraquat (methyl viologen hydrate, Fisher Scientific, Hampton, NH) was dissolved in water to a concentration of 3–5 mM. Paraquat solution was either stored at 4°C for up to 1 month or frozen in aliquots and thawed as needed. For every experimental genotype treated with paraquat injection, we conducted mock injections with ddH_2_O to control for wounding sensitivity (S4 Fig).

### H_2_O_2_ feeding assays

These assays were performed in two ways. In one method, flies were transferred to vials containing a folded Kimwipe soaked with 1.5 mL of a 5% sucrose, 1%–4% H_2_O_2_ solution. Thirty percent H_2_O_2_ (Sigma-Aldrich, St. Louis, MO) was diluted in ddH_2_O to a concentration of 1%–4% depending on the death rate for the given genotype, titrated to complete death within several days. Flies were flipped onto a freshly soaked Kimwipe every 2 days and death was assayed visually and recorded daily. This method allows very rapid setup (typical experiment used 40 flies/genotype) but provides relatively low-resolution survival kinetics. In the second method, flies were transferred to 5 mm tubes containing a piece of a soaked Kimwipe and loaded into *Drosophila* Activity Monitors, in which case death was determined by a complete loss of movement. Control flies were kept on 5% sucrose alone to ensure that death did not occur by starvation or desiccation. This method provides high-resolution survival kinetics but requires more time-intensive setup (typical experiment used 30 flies/genotype). We found that all our results for short-sleeping mutants were consistent between the two methods.

### Survival curves

Survival curves for starvation assays, lifespan experiments, bacterial infections, paraquat injections, and H_2_O_2_ feeding assays are all plotted as Kaplan-Meier graphs. Log-rank analysis was performed using GraphPad Prism (GraphPad Software, La Jolla, CA). All experiments were performed with a minimum of three independent trials and yielded statistically similar results, except where noted. Graphs and *p*-values in figures are from representative trials.

### qRT-PCR

Age-matched, 6–8-day-old flies were anesthetized on ice and decapitated between ZT2 and ZT5. Fifteen to twenty heads per sample were homogenized in TRIzol (Invitrogen), and a phenol-chloroform extraction was performed to isolate nucleic acids. Samples were treated with DNAse (Invitrogen, Carlsbad, CA) to isolate RNA and then diluted to a concentration of about 60 ng/μL. RevertAid First Strand cDNA synthesis kit (ThermoFisher, Waltham, MA) was used to convert RNA to cDNA. Quantitative RT-PCR was performed using a Bio-Rad CFX Connect Real-Time qPCR machine, with Express Sybr GreenER qPCR SuperMix (Invitrogen, Carlsbad, CA) and the following primer sets:

*SOD1*:

For: GGAGTCGGTGATGTTGACCT

Rev: GGAGTCGGTGATGTTGACCT

*GSTS1*:

For: CACCAGAGCATTTCGATGGCT

Rev: ACGACTGCAATTTTTAGACGGA

*GSTO1*:

For: ACGACTGCAATTTTTAGACGGA

Rev: CCGATCGCCGGGAGTTCATGTAT

*catalase*:

For: TTCTGGTTATCCCGTTGAGC

Rev: GGTAATGGCACCAGGAGAAA

*hsp60*:

For: TGATGCTGATCTCGTCAAGC

Rev: TACTCGGAGGTGGTGTCCTC

*ClpX*:

For: AAAATGCTCGAAGGCACAGT

Rev: TTGAGACGACGTGCGATAAG

Pink1:

For: TCGGTGGTCAATGTAGTGC

Rev: CCACTCGGAAGATTCCACTGC

*BiP*:

For: GCTATTGCCTACGGTCTGGA

Rev: CATCACACGCTGATCGAAGT

*actin*:

For: TTGTCTGGGCAAGAGGATCAG

Rev: ACCACTCGCACTTGCACTTTC

Analysis was performed using the Standard Curve method. Total cDNA concentration was normalized to actin expression. Data are represented as mean ± SEM. Five to six biological replicates (containing 15–20 heads each) per experiment.

### Gaboxadol and antioxidant feeding

Gaboxadol hydrochloride (Sigma-Aldrich, St. Louis, MO) was dissolved in water and added to melted cornmeal food to a final concentration of 0.1–0.2 mg/mL. Flies were flipped onto Gaboxadol-containing food for 3 days prior to paraquat injection and remained on Gaboxadol-containing food postinjection. Control food was made by adding the appropriate amount of vehicle alone (H_2_O) to melted cornmeal food.

## Supporting information

S1 Fig Neuronal *inc-RNAi* flies and *fumin* mutants are short sleeping and do not display a global immunity defect. (A) Twenty-four-hour sleep plot for neuronal *inc-RNAi* flies and controls. Neuronal *inc-RNAi* flies died at the same or a slightly slower rate than genetic controls after injection with *Listeria monocytogenes* (B, *p* = 0.09 compared to *elav* control, *p* = 0.04 compared to *inc-RNAi* control, *n* = 62–63 flies/genotype) and died at the same rate as controls after injection with *Staphylococcus aureus* (C, *p* > 0.05 compared to either control, *n* = 19–21 flies/genotype). (D) Twenty-four-hour sleep plot for *fumin* mutants and controls. *fumin* mutants died slower than controls after injection with *Streptococcus pneumoniae* (E, *p* < 0.01, *n* = 96–98 flies/genotype), died faster than controls after injection with *Providencia rettgeri* (F, *p* < 0.0001, *n* = 89–91 flies/genotype), died slower than controls after injection with *L*. *monocytogenes* (G, *p* < 0.01, *n* = 77–79 flies/genotype), and died at the same rate as controls after injection with *S*. *aureus* (H, *p* > 0.05, *n* = 94–100 flies/genotype). *p*-values were obtained by log-rank analysis. Data from representative experiments are shown. Each experiment was performed at least three times. Raw data from representative experiments are available in S1 Data; raw data from all trials are available upon request. *inc*, *insomniac*; *RNAi*, RNA interference.
(TIF)

S2 Fig Reduction of *inc or Cul3* causes short sleep. *inc*^1^ and *inc*^2^ null mutants slept about 50% less than controls (A, *p* < 0.0001 for both mutants, *n* = 20–22 flies/ genotype). *elav;;dcr>Cul3-RNAi* flies slept about 60% less than controls (B, *p* < 0.0001 compared to either control, *n* = 40–42 flies/genotype). Each data point in scatterplots (left) represents average sleep in minutes/day measured across 4–5 days in an individual animal. Data are shown as mean ± SEM. *p*-values were obtained by ordinary one-way ANOVA followed by a post hoc Tukey test. Twenty-four-hour sleep plots (right) show sleep profiles for mutants and controls averaged over a 4–5-day period. Data from representative experiments are shown. Each experiment was performed at least three times. Raw data from representative experiments are available in S1 Data; raw data from all trials are available upon request. *Cul3*, Cullin-3; *dcr*, UAS-Dicer; *inc*, *insomniac*; *RNAi*, RNA interference.
(TIF)

S3 Fig Twenty-four-hour sleep plots for short-sleeping mutants. Shown here are the 24-hour sleep plots, averaged over 4–5 days, for the indicated short-sleeping flies, with their relevant controls. (A) *sleepless* mutants and controls; relates to Fig 3B. (B) *redeye* mutants and controls; relates to Fig 3D. (C) *dFB>NaChBac* flies and controls; relates to Fig 4A. (D) Gaboxadol-fed flies compared with vehicle only; relates to Fig 4B. (E–G) Neuronal overexpression of *Catalase*, *SOD1*, and *SOD2*, compared with controls; relates to Fig 6. Raw data from representative experiments are available in S1 Data; raw data from all trials are available upon request. *dFB*, dorsal Fan-shaped Body; *SOD*, *superoxide dismutase*.
(TIF)

S4 Fig Paraquat injection controls. Shown here are representative H_2_O-injected wounding controls for each of the genotypes subjected to paraquat injection: (A) neuronal *inc-RNAi* (relates to Fig 2B); (B) *inc* null mutants (relates to Fig 2C); (C) neuronal *Cul3-RNAi* (relates to Fig 2D); (D) *sleepless* mutants (relates to Fig 3B); (E) *fumin* mutants (relates to Fig 3C); (F) *redeye* mutants (relates to Fig 3D); (G) *dFB>NaChBac* flies (relates to Fig 4A); and (H) *iso31* controls (relates to Fig 4B). In all cases, flies injected with paraquat died significantly faster (*p* < 0.5 by log-rank analysis) than H_2_O-injected controls. Raw data from representative experiments are available in S1 Data; raw data from all trials are available upon request. *Cul3*, Cullin-3; *dFB*, dorsal Fan-shaped Body; *inc*, *insomniac*; *RNAi*, RNA interference.
(TIF)

S1 Table Data summary. Summary of results from experimental trials. Raw data from representative experiments are available in S1 Data; raw data from all trials are available upon request.
(XLSX)

S1 Data Raw data from representative experiments. Raw data from representative experiments are organized here by figure panel; raw data from all trials are available upon request.
(XLSX)

## Abbreviations

BHI: Brain Heart Infusion media
Cul3: Cullin-3
DAT: dopamine transporter
dcr: Dicer
dFB: dorsal Fan-shaped Body
GST: glutathione-S-transferase
H_2_O_2_: hydrogen peroxide
inc: insomniac
nAChR: nicotinic acetylcholine receptor
RNAi: RNA interference
ROS: reactive oxygen species
SEM: standard error of the mean
UAS: upstream activation sequence
qRT-PCR: quantitative reverse transcription polymerase chain reaction
SOD: superoxide dismutase
