# Supplementary figures and images for: A bidirectional relationship between sleep and oxidative stress in Drosophila
Source: PLoS Biol. 2018 Jul 12;16(7):e2005206. doi: 10.1371/journal.pbio.2005206 (PMC6042693; doi:10.1371/journal.pbio.2005206)

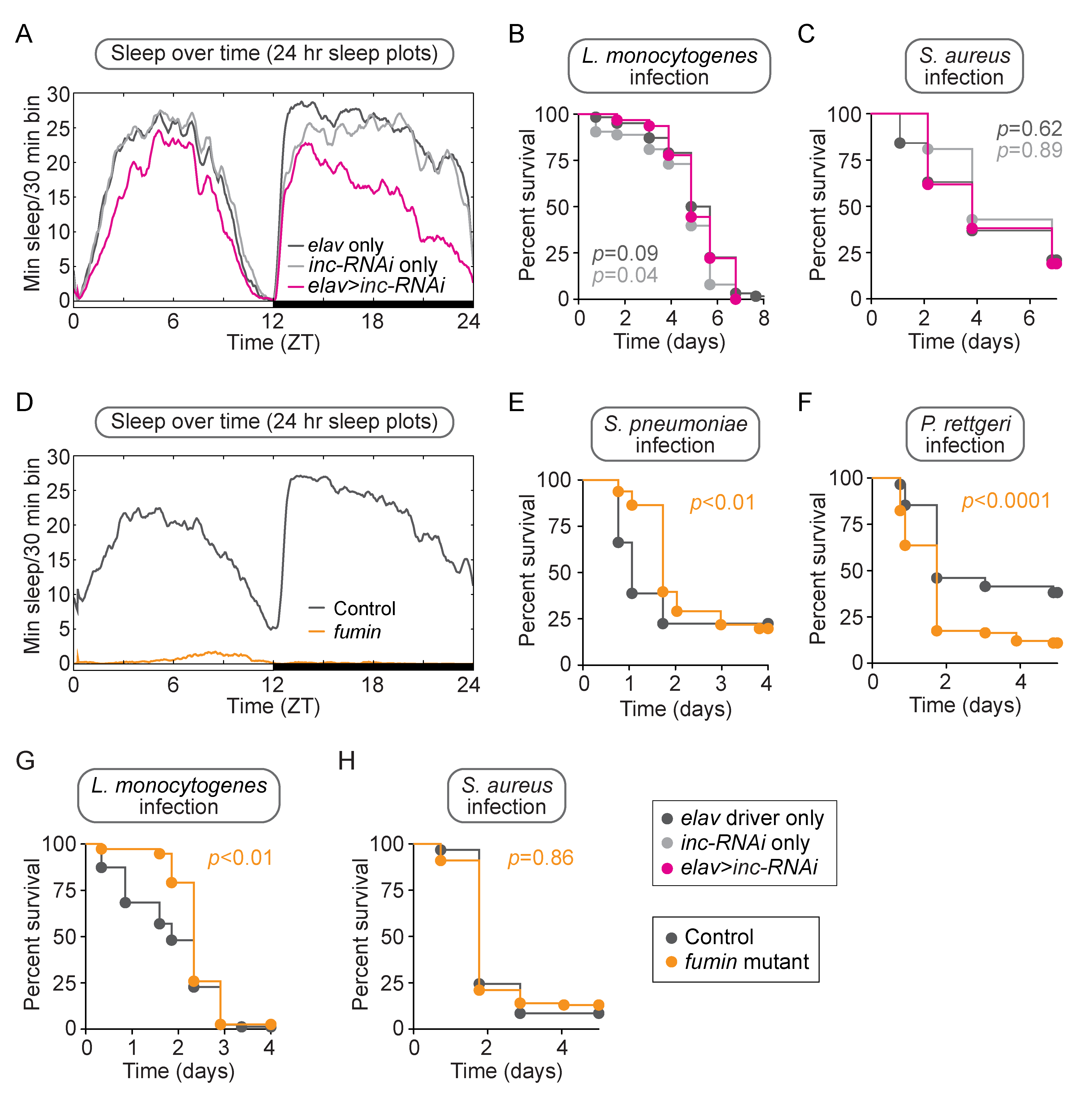

Supplement: S1 Fig — (A) Twenty-four-hour sleep plot for neuronal inc-RNAi flies and controls. Neuronal inc-RNAi flies died at the same or a slightly slower rate than genetic controls after injection with Listeria monocytogenes (B, p = 0.09 compared to elav control, p = 0.04 compared to inc-RNAi control, n = 62–63 flies/genotype) and died at the same rate as controls after injection with Staphylococcus aureus (C, p > 0.05 compared to either control, n = 19–21 flies/genotype). (D) Twenty-four-hour sleep plot for fumin mutants and controls. fumin mutants died slower than controls after injection with Streptococcus pneumoniae (E, p < 0.01, n = 96–98 flies/genotype), died faster than controls after injection with Providencia rettgeri (F, p < 0.0001, n = 89–91 flies/genotype), died slower than controls after injection with L. monocytogenes (G, p < 0.01, n = 77–79 flies/genotype), and died at the same rate as controls after injection with S. aureus (H, p > 0.05, n = 94–100 flies/genotype). p-values were obtained by log-rank analysis. Data from representative experiments are shown. Each experiment was performed at least three times. Raw data from representative experiments are available in S1 Data; raw data from all trials are available upon request. inc, insomniac; RNAi, RNA interference. (TIF) [file pbio.2005206.s001.tif]

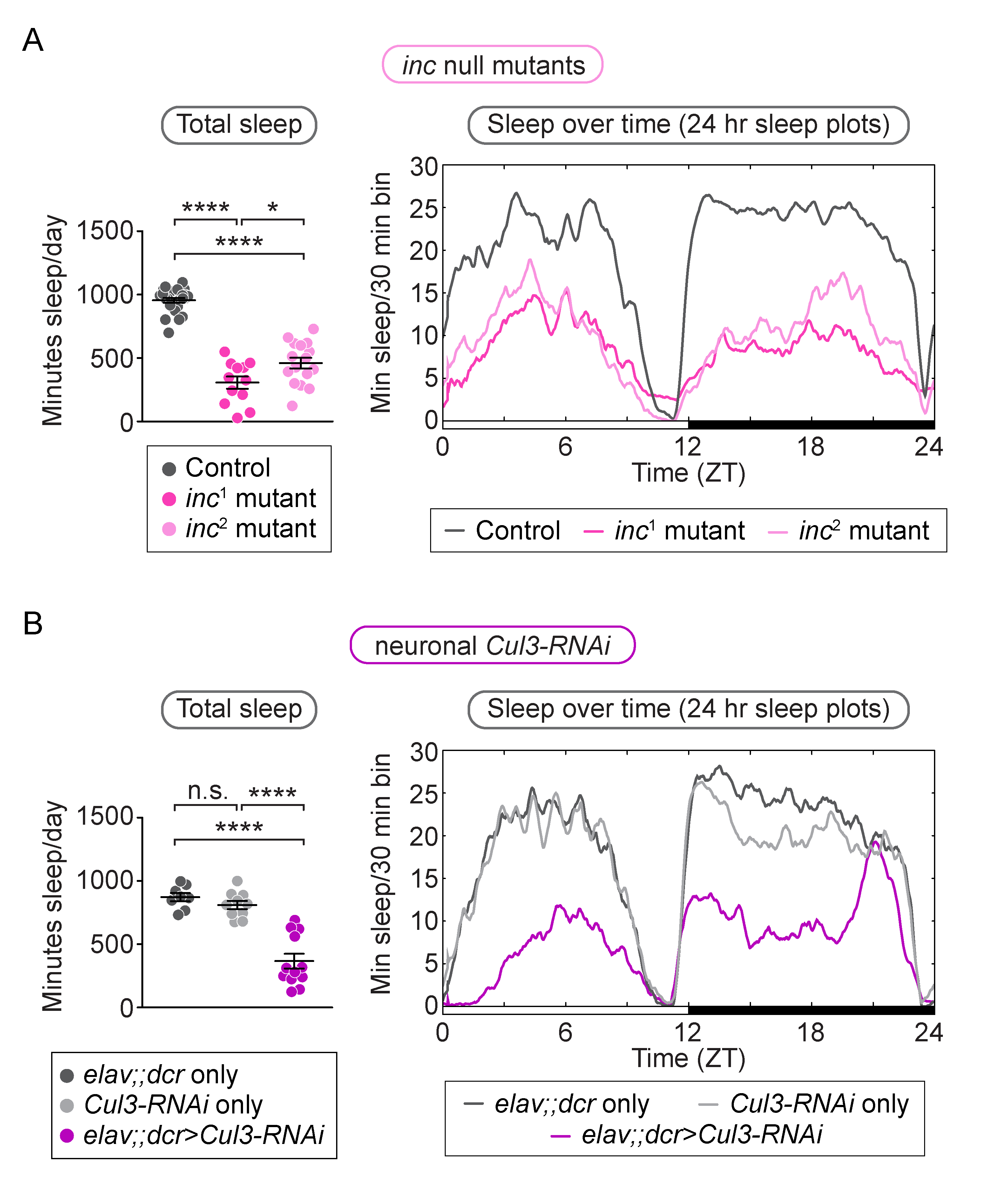

Supplement: S2 Fig — inc1 and inc2 null mutants slept about 50% less than controls (A, p < 0.0001 for both mutants, n = 20–22 flies/ genotype). elav;;dcr>Cul3-RNAi flies slept about 60% less than controls (B, p < 0.0001 compared to either control, n = 40–42 flies/genotype). Each data point in scatterplots (left) represents average sleep in minutes/day measured across 4–5 days in an individual animal. Data are shown as mean ± SEM. p-values were obtained by ordinary one-way ANOVA followed by a post hoc Tukey test. Twenty-four-hour sleep plots (right) show sleep profiles for mutants and controls averaged over a 4–5-day period. Data from representative experiments are shown. Each experiment was performed at least three times. Raw data from representative experiments are available in S1 Data; raw data from all trials are available upon request. Cul3, Cullin-3; dcr, UAS-Dicer; inc, insomniac; RNAi, RNA interference. (TIF) [file pbio.2005206.s002.tif]

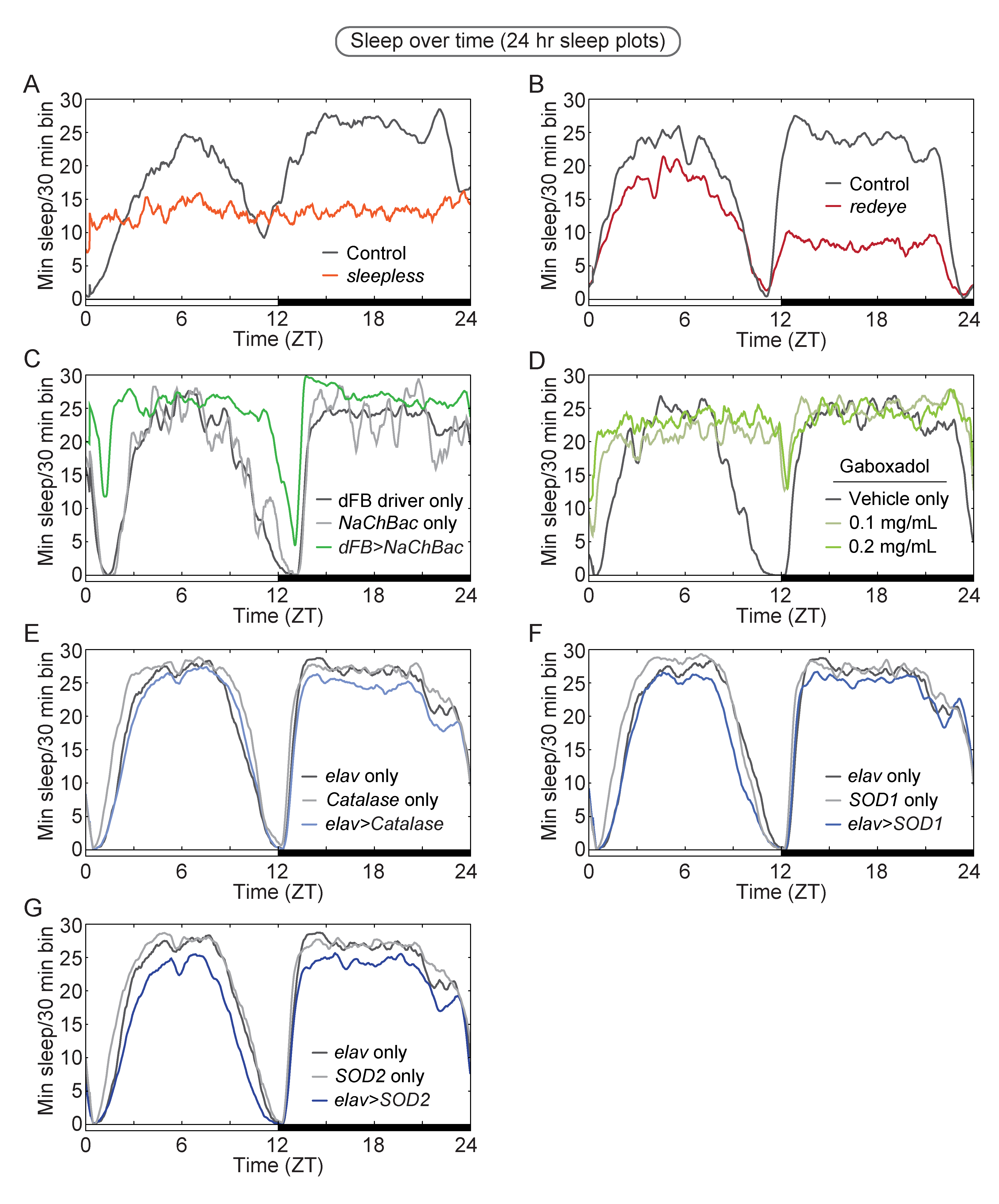

Supplement: S3 Fig — Shown here are the 24-hour sleep plots, averaged over 4–5 days, for the indicated short-sleeping flies, with their relevant controls. (A) sleepless mutants and controls; relates to Fig 3B. (B) redeye mutants and controls; relates to Fig 3D. (C) dFB>NaChBac flies and controls; relates to Fig 4A. (D) Gaboxadol-fed flies compared with vehicle only; relates to Fig 4B. (E–G) Neuronal overexpression of Catalase, SOD1, and SOD2, compared with controls; relates to Fig 6. Raw data from representative experiments are available in S1 Data; raw data from all trials are available upon request. dFB, dorsal Fan-shaped Body; SOD, superoxide dismutase. (TIF) [file pbio.2005206.s003.tif]

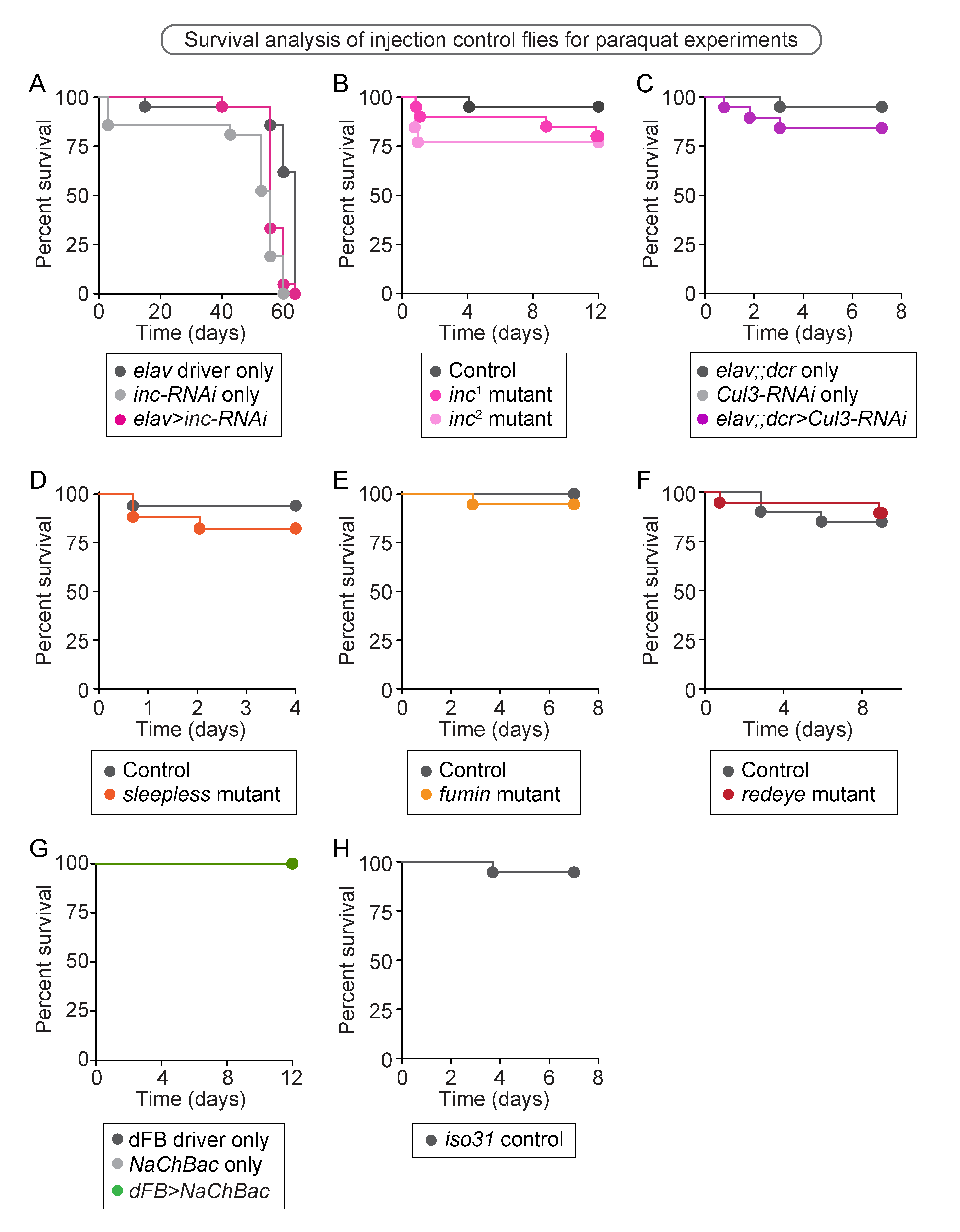

Supplement: S4 Fig — Shown here are representative H2O-injected wounding controls for each of the genotypes subjected to paraquat injection: (A) neuronal inc-RNAi (relates to Fig 2B); (B) inc null mutants (relates to Fig 2C); (C) neuronal Cul3-RNAi (relates to Fig 2D); (D) sleepless mutants (relates to Fig 3B); (E) fumin mutants (relates to Fig 3C); (F) redeye mutants (relates to Fig 3D); (G) dFB>NaChBac flies (relates to Fig 4A); and (H) iso31 controls (relates to Fig 4B). In all cases, flies injected with paraquat died significantly faster (p < 0.5 by log-rank analysis) than H2O-injected controls. Raw data from representative experiments are available in S1 Data; raw data from all trials are available upon request. Cul3, Cullin-3; dFB, dorsal Fan-shaped Body; inc, insomniac; RNAi, RNA interference. (TIF) [file pbio.2005206.s004.tif]
